# Towards a Whole Sample Imaging Approach Using Diffusion Tensor Imaging to Examine the Foreign Body Response to Explanted Medical Devices

**DOI:** 10.3390/polym14224819

**Published:** 2022-11-09

**Authors:** Ruth E. Levey, Brooke Tornifoglio, Alan J. Stone, Christian Kerskens, Scott T. Robinson, Fergal B. Coulter, Robert Bagnall, Raymond O’Connor, Eimear B. Dolan, Peter Dockery, Gabriella Bellavia, Stefania Straino, Francesca Cianfarani, Paul Johnson, Eoin O’Cearbhaill, Caitríona Lally, Garry P. Duffy

**Affiliations:** 1 Discipline of Anatomy & Regenerative Medicine Institute, School of Medicine, College of Medicine, Nursing and Health Sciences, University of Galway, H91 W5P7 Galway, Ireland; 2 Trinity Centre for Biomedical Engineering, Trinity Biomedical Science Institute, Trinity College Dublin, D02 R590 Dublin, Ireland; 3 Department of Mechanical, Manufacturing and Biomedical Engineering, School of Engineering, Trinity College Dublin, D02 R590 Dublin, Ireland; 4 Department of Medical Physics and Clinical Engineering, St. Vincent’s University Hospital Dublin, D04 T6F4 Dublin, Ireland; 5 Trinity College Institute of Neuroscience, Trinity College Dublin, D02 R123 Dublin, Ireland; 6 UCD Centre for Biomedical Engineering, School of Mechanical and Materials Engineering, University College Dublin, D04 R7R0 Dublin, Ireland; 7 Department of Biomedical Engineering, School of Engineering, College of Science and Engineering, University of Galway, H91 HX31 Galway, Ireland; 8 Preclinical Unit, Explora Biotech Srl, G. Peroni 386, 00131 Rome, Italy; 9 Nuffield Department of Surgical Sciences and NIHR Biomedical Research Centre, Oxford Centre for Diabetes Endocrinology and Metabolism, University of Oxford, Oxford OX3 9DU, UK; 10 Advanced Materials and Bioengineering Research Centre (AMBER), Royal College of Surgeons in Ireland and Trinity College Dublin, D02 YN77 Dublin, Ireland

**Keywords:** foreign body response, fibrous capsule, diffusion tensor imaging, macroencapsulation

## Abstract

Analysing the composition and organisation of the fibrous capsule formed as a result of the Foreign Body Response (FBR) to medical devices, is imperative for medical device improvement and biocompatibility. Typically, analysis is performed using histological techniques which often involve random sampling strategies. This method is excellent for acquiring representative values but can miss the unique spatial distribution of features in 3D, especially when analysing devices used in large animal studies. To overcome this limitation, we demonstrate a non-destructive method for high-resolution large sample imaging of the fibrous capsule surrounding human-sized implanted devices using diffusion tensor imaging (DTI). In this study we analyse the fibrous capsule surrounding two unique macroencapsulation devices that have been implanted in a porcine model for 21 days. DTI is used for 3D visualisation of the microstructural organisation and validated using the standard means of fibrous capsule investigation; histological analysis and qualitative micro computed tomography (microCT) and scanning electron microscopy (SEM) imaging. DTI demonstrated the ability to distinguish microstructural differences in the fibrous capsules surrounding two macroencapsulation devices made from different materials and with different surface topographies. DTI-derived metrics yielded insight into the microstructural organisation of both capsules which was corroborated by microCT, SEM and histology. The non-invasive characterisation of the integration of implants in the body has the potential to positively influence analysis methods in pre-clinical studies and accelerate the clinical translation of novel implantable devices.

## 1. Introduction

No medical device is immune to its fate of being, to some extent, encapsulated as a result of the foreign body response (FBR). This response is immune-mediated, whereby a foreign body or implanted material stimulates a plethora of inflammatory events and wound-healing processes, causing cellular and collagenous deposition, and often the formation of a dense fibrous capsule surrounding the implant [1,2,3]. This collagenous encapsulation can compromise the performance and endurance of the medical implant by impairing biosensing capabilities, causing pain, isolating cell-based implants from vascularisation and nourishment, hindering drug elution, and eventually causing implant/device failure [1,2,3]. Overcoming and understanding the FBR to different biomaterials is extremely important and therefore in-depth examinations following pre-clinical studies is essential to analyse the composition and organisation of the fibrous capsule, as these features ultimately forecast the possible success or failure of an implant. Our research predominantly focusses on islet cell transplantation via novel macroencapsulation devices and we have demonstrated the transition from small to large animal studies which will ultimately pave the way to proceed to Phase I and II of clinical trials in human subjects [4,5]. 

Currently the standard practice for fibrous capsule examination lies in histological analysis. This approach for evaluating the microstructure of the fibrous capsule often relies on random sampling. This method is excellent for acquiring representative measurements; however, cannot account for the unique spatial distribution of features in 3D. Not to mention, this method can often require large numbers of animals for accurate analysis and additional qualitative imaging. All this becomes even more difficult when analysing human-sized devices used in large animal studies. Due to the fact that samples this large cannot be processed for histology, more sampling is required; however, this only provides a minute snapshot of the capsule composition at random locations. For these reasons, a high-resolution, non-invasive, volumetric imaging technique for large tissue samples would be extremely beneficial in the characterisation of the fibrous capsule. 

While existing non-invasive imaging techniques, such as ultrasound and fluorescence imaging [6,7,8], can probe for information regarding inflammation which would be relevant for monitoring the FBR, these methods lack any further microstructural information on the fibrous capsule. Diffusion tensor imaging (DTI) is a magnetic resonance imaging technique which allows for characterisation of the underlying microstructure within a tissue by exploiting the diffusion of water [9]. While predominantly developed for use in the brain, DTI has recently been applied both ex vivo [10,11,12,13] and in vivo [14,15,16] to a multitude of extracranial locations in order to gain an insight into the morphologies of healthy and diseased tissues. The application of DTI to examine the fibrous capsule has yet to be explored; however, it has been used to investigate myocardial [17] and caesarean [18] scar tissue, which are similar in composition to the fibrous capsule. Currently, qualitative MRI approaches, such as T1- and T2-weighted imaging, are used to anatomically identify the presence of a fibrous capsule around silicone breast implants [19]; however, DTI offers the potential to not only qualitatively identify the presence of a fibrous capsule but also yields parametric maps of mean diffusivity (MD) and fractional anisotropy (FA) capable of quantifying microstructural organisation.

In this study, we investigate the potential of DTI to inform on the presence, or lack-there-of, of the formation of a fibrous capsule, as well as its organisation. We describe high-resolution DTI for imaging explanted tissue surrounding two unique large scale devices which have been implanted in a porcine model for 21 days. DTI allows for 3D visualisation of the tissue microstructure and is validated using the standard means of fibrous capsule investigation: histological analysis and qualitative microCT and SEM imaging. DTI offers a promising non-invasive, non-destructive imaging modality that can yield novel insight into the formation of fibrous capsule and tissue-device integration.

## 2. Materials and Methods

### 2.1. Device Fabrication & Animal Study

Human sized macroencapsulation devices were produced as previously described [4,5]. Porcine studies were approved by the Italian Ministry of Health (Authorisation No. 976/2017-PR). Four female Landrace pigs, weighing 25–30 kg were enrolled in the study. Devices were implanted in the anterior abdominal wall as previously described [4,5] *n* = 2 per group. Following euthanasia, both devices were removed en bloc with surrounding muscle tissue and fixed in 4% PFA for 48 h.

### 2.2. MR Imaging and Analysis 

Fixed devices were stored at 4 °C in 70% ethanol until MR imaging. All samples were placed in a 3D printed holder made from polylactic acid custom made to fit the MRI coil. For all imaging the holder was filled with fresh PBS at room temperature and devices were imaged individually at room temperature (approx. 24 °C). A 7T Bruker BioSpec 70/30 USR system (Bruker, Ettinger, Germany) equipped with Paravision 6 (Bruker, Ettinger, Germany) and a volume coil were used for all imaging sequences. T1- and T2-weighted scans were used to visualize the device and the surrounding tissue. T1-weighted parameters were as follows: TE/TR: 7/1452 ms, flip angle: 40°, 20 averages, image size: 256 × 256 × 100, field of view: 70 × 70 × 50 mm, resolution: 0.273 × 0.273 × 0.5 mm and acquisition time: 1 h and 23 min. T2-weighted parameters were as follows: TE/TR: 19.55/9137.6 ms, 20 averages, echo spacing: 6.518 ms, RARE factor: 8, image size: 256 × 256 × 30, field of view: 70 × 70 × 15 mm, resolution: 0.273 × 0.273 × 0.5 mm, and acquisition time: 1 h and 13 min. A 2D spin echo DTI sequence was used for all four samples. One multiscale porosity device was imaged with the following parameters: TE/TR: 18.182/1011 ms, 5 averages, image size: 128 × 128 × 47, field of view: 64 × 64 × 23.5 mm, isotropic resolution: 0.5 × 0.5 × 0.5 mm, b-values: 0, 800 s/mm^2^, 32 diffusion directions, gradient duration: 3.8 ms, gradient separation: 8.802 ms and acquisition time 5 h and 55 min. The second multiscale porosity device and the two smooth devices were imaged with a refined 2D DTI sequence with the following parameters: TE/TR: 18.182/4000 ms, 4 averages, image size: 140 × 140 × 20, field of view: 70 × 70 × 10 mm, isotropic resolution 0.5 × 0.5 × 0.5 mm, acquisition time of 20 h and 32 min and the same diffusion parameters. While the SNR of the refined 2D DTI sequence was higher than the initial sequence, the SNR ratio between B0:B800 was comparable between sequences.

Prior to calculation of the diffusion tensor, all raw data was denoised (‘dwidenoise’) and corrected for Gibbs ringing (‘mrdegibbs’) using MRtrix3 (Melbourne, Australia, http://github.com/MRtrix3/mrtrix3, accessed on 29 August 2022). The diffusion tensor was then estimated using the open-source software ExploreDTI (Utrecht, The Netherlands, www.exploredti.com, accessed on 29 August 2022) where the fractional anisotropy (FA) and mean diffusivity (MD) were calculated. Regions of interest (capsule and tissue) were defined based on the T1- and T2-weighted scans. Mean-diffusion weighted images were obtained by averaging the 32 diffusion-weighted images. The capsule was identified as the tissue adjacent to the device while muscle tissue not near the device was simply termed ‘tissue’. Mean values for FA and MD are reported. Normalised FA and MD values were calculated by dividing the metric of interest in the capsule by the value in the tissue of the same device. Tractography was done in ExploreDTI and parameters were kept consistent between all devices and are as follows: seed point resolution: 0.5 × 0.5 × 0.5 mm, FA threshold: 0.1, FA tracking threshold: 0.1–1, tract length: 2–20 mm, angular threshold: 30° and step size: 0.5 mm. 

### 2.3. Tissue Processing and Histology 

Core biopsy samples were taken systematically at five locations across each device using an 8 mm punch biopsy and placed in a 2% agarose to maintain structure. Four of these cores were for histological analysis while the 5th was processed for SEM imaging. Cores for histological analysis were processed and embedded in paraffin wax blocks. Sections of 5 μm were cut and stained with Masson’s Trichrome with Gomori’s Aldehyde Fuchsin, picrosirius red and αSMA and imaged for fibrous capsule analysis as detailed previously [5,20] Quantitative analysis of the collagen content for coherency and relative integrated colour densities was carried out on picrosirius red stained sections using a previously reported technique [5,20,21,22].

### 2.4. Scanning Electron Microscopy (SEM) 

Each core was bisected longitudinally and prepared as detailed by Coulter et al. [5]. A Quorum Q150R ES plus (Sussex, UK) was used to lightly sputter coat the samples. Specimens were imaged using a Hitachi S2600N Scanning Electron Microscope (Krefeld, Germany) using a secondary electron detector (Vacuum 15 kV, electron Beam 50, resolution 1280 × 960 PPI). SEM images were pseudo-coloured using MountainsMap^®^ SEM Color 7.3.7984 (Besançon, France) 

### 2.5. MicroCT

Whole tissue samples were stained with 2.5% PMA solution in 70% ethanol for 7 days, then washed, and stored in fresh 70% ethanol. A microCT 100 scanner (Scanco) at 70 kVp and 85 μA with a 0.5 mm aluminum filter was used. MicroCT DICOM files were segmented using Mimics Research 18.0.0.525 software (Materialise) as described previously [20].

### 2.6. Statistical Methods

Statistical analysis was performed with GraphPad Prism (Version 8). Nonparametric tests were performed as *n* < 2 per group. Kruskal–Wallis tests with Dunn’s multiple comparisons were performed on mean FA and MD values per sample. Mann–Whitney U tests were performed on PLM analysis of coherency and relative integrated density of coloured fibres. *n* = 2 for both the multiscale porosity and smooth devices. Mean values are reported and error bars represent standard deviation.

## 3. Results

### 3.1. In Vivo Implantation of Macroencapsulation Devices

**Figure 1:**
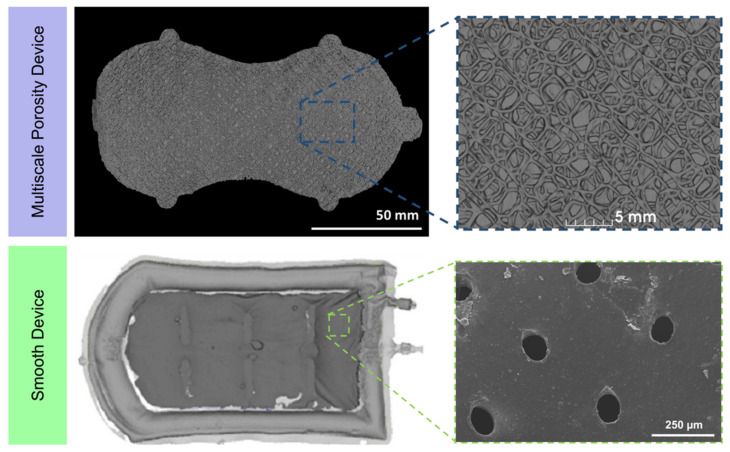
Volumetric renderings of devices after microCT imaging with associated high magnification images demonstrating both multiscale porosity surface [5] and smooth surface [4], respectively.

In this study we examined two novel human-sized macroencapsulation devices that were implanted in the anterior abdominal wall of a diabetic porcine model for 21 days. Devices and the surrounding tissue were carefully explanted and fixed for 48 h. These implantations were part of larger clinical translation studies [4,5]; however, for the purpose of this paper, they were used to demonstrate the potential of DTI as an investigative tool for fibrous capsule composition. Both devices were unique in their polymer composition, porosity and topography (Figure 1). The first device, the “Multiscale Porosity Device”, was composed of silicone, with both microporosity and macroporosity, in order to promote cellular attachment and tissue integration, respectively. The second device, the “Smooth Device”, was composed of a soft, flexible thermoplastic polymer (thermoplastic polyurethane, or TPU). Its surface was smooth. By investigating the tissue around these starkly different device topographies with DTI, we established the sensitivity of this imaging modality to characterise the tissue microstructure surrounding implanted devices. 

### 3.2. Multi-Contrast MRI

**Figure 2:**
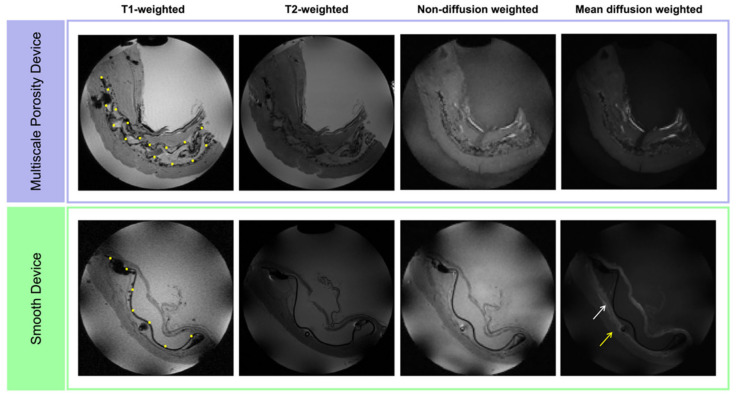
Multi-contrast MRI images of both multiscale porosity and smooth devices. T1- and T2-weighted and non-diffusion weighted (b = 0 s/mm²) images showed little contrast within the samples, while the mean diffusion weighted images (b = 800 s/mm², 32 directions) show contrast between the tissue around the smooth device (white arrow) and the more distal tissue (yellow arrow). Representative slices are shown, yellow dots mark the device location.

Standard anatomical T1- and T2-weighted imaging was used to identify the implanted device. While the devices were identifiable, little distinguishable contrast was seen between the tissue surrounding the device and more distally located muscular tissue (Figure 2). However, when looking at the mean diffusion weighted images (b = 800 s/mm^2^, 32 directions) of both devices, the tissue surrounding the smooth device showed distinct contrast to the tissue more distally located from the device.

### 3.3. DTI Metrics 

**Figure 3:**
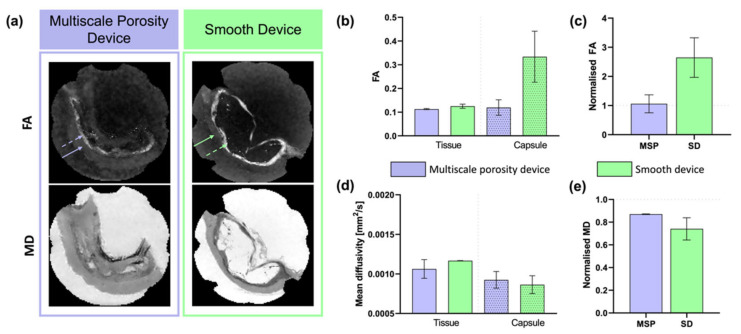
DTI metrics for both devices. (**a**) Fractional anisotropy (top) and mean diffusivity (bottom) maps for both devices. Scales of images match the scales in (**b**,**d**), respectively. Solid arrows point to tissue regions and dashed arrows to the capsule. (**b**,**d**) Fractional anisotropy and mean diffusivity shown for the surrounding tissue and the tissue-device boundary (capsule). Capsule regions were normalised by the respective tissue regions per sample to give normalised (**c**) fractional anisotropy and (**e**) mean diffusivity values. A value of 1 indicates no difference between the capsule and surrounding tissue. *n* = 2 per group; data are represented as means ± standard deviation. No significant difference was determined using Kruskal–Wallis tests. MSP—multiscale porosity device, SD—smooth device.

Figure 3 presents DTI metrics for the two devices. Parametric maps of FA (Figure 3a, top row) showed the stark difference in capsule delineation between devices; the regions of elevated FA surrounding the smooth devices highlight a highly anisotropic tissue around the device. Similarly, the MD maps (Figure 3a, bottom row) showed decreased diffusivity in the same region. Quantitatively, the mean FA in the tissue and capsule regions for the multiscale porosity device were 0.11 ± 0.0021 and 0.12 ± 0.033, respectively. For the smooth device the mean FA in the tissue was similar to the multiscale porous device at 0.12 ± 0.0086 but the capsule was much more anisotropic with an FA of 0.33 ± 0.11. The MD in the tissue regions for the multiscale porosity and smooth device, 1.1 ± 0.12 × 10^−3^ mm^2^/s and 1.2 ± 0.0019 × 10^−3^ mm^2^/s, were similar and also compared well with the MD in the capsule region for the multiscale porosity device, 0.93 ± 0.11 × 10^−3^ mm^2^/s. This was in stark contrast to the notably lower diffusivity in the fibrous capsule of the smooth device which had an MD of 0.86 ± 0.11 × 10^−3^ mm^2^/s. In order to ascertain differences between the tissue surrounding the device and tissue more distally located from the device, the capsule regions were normalised by these distal muscular tissue regions (Figure 3c). The farther away from 1 the value was, the larger the difference between the two regions. From this, it became even more evident just how different the tissue around the smooth device was from the surrounding tissue. The elevated normalised FA in the smooth device highlighted a higher alignment while the decreased normalised MD showed decreased diffusivity in the capsule compared to the normal tissue.

**Figure 4:**
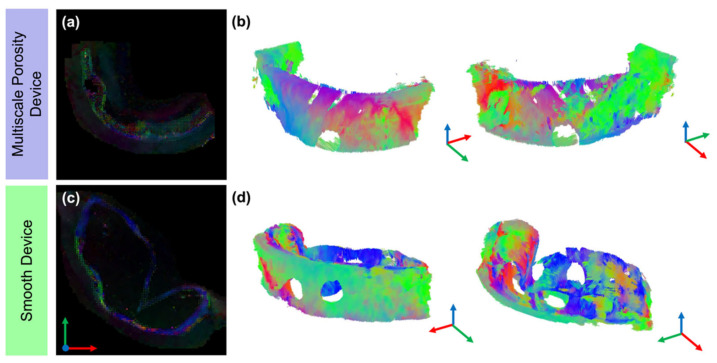
Vector maps and tractography for both devices. (**a**,**c**) First eigenvector-fractional anisotropy maps for both devices. (**b**,**d**) Tractography for the multiscale porosity device and smooth device, respectively. No clear delineation of a capsule is visible in the multiscale porosity device while the alignment of a capsule is clear in the smooth device. Holes are locations of biopsy punches for other analyses. Tractography was modelled with the following parameters: seed point resolution: 0.5 × 0.5 × 0.5 mm, FA threshold: 0.1, FA tracking threshold: 0.1–1, tract length: 2–20 mm, angular threshold: 30° and step size of 0.5 mm.

First eigenvector-fractional anisotropy (FEFA) maps are shown in Figure 4a,c; where the colour coding indicates the principal direction of diffusion and the intensity is weighted by the fractional anisotropy. The FEFA map for the multiscale porosity device (Figure 4a) showed no coherent principal direction around the device, while the FEFA map of the smooth device illustrated a predominantly axial (blue) direction of diffusion. Tractography was performed to better visualise the diffusion pathways and microstructure between the capsule and surrounding tissue. Keeping the tracking parameters consistent, Figure 4b,d show 3D representations of the microstructural arrangement. In the multiscale porosity device, no tracts were modelled around the device but instead only in the muscular tissue. Meanwhile, in the smooth device the capsule was clearly modelled at the tissue-device boundary. The blue tracts (Figure 4d) highlight the longitudinal alignment in the capsule on the top and bottom of the device, while around the curvature of the lateral edges of the device a more circumferential alignment can be seen.

### 3.4. MicroCT & SEM Imaging

**Figure 5:**
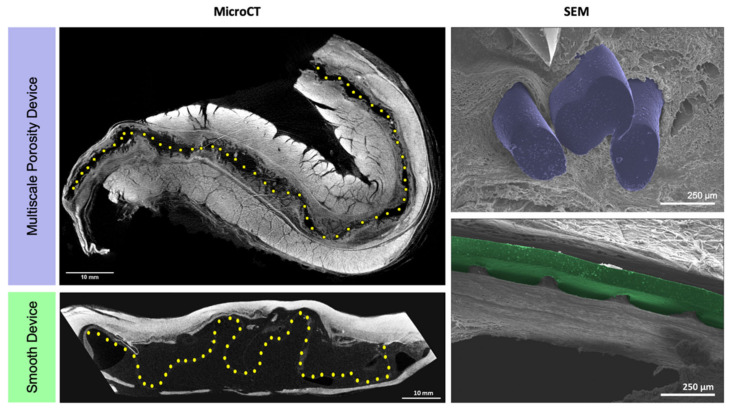
MicroCT cross sectional images (**left** column) of multiscale porosity and smooth surfaced devices, demonstrating their relationship with the surrounding tissue. Yellow dots are indicative of device location. Scale bars = 10 mm. High magnification SEM images (**right** column) of rope-coil surface features (blue) integrated into the surrounding tissue and reduced integration surrounding the smooth device (green). Scale bar = 250 μm.

MicroCT & SEM imaging was performed to gain insight into both the organisation of the fibrous capsules and also the relationship between the device topography and the surrounding tissue. The impact of the multiscale porosity devices’ surface features on the integration with the immediate surrounding tissue were strikingly apparent in the cross-sectional images from microCT (Figure 5). Meanwhile, the smooth device showed decreased integration due to lack of surface features acting as a scaffold for the surrounding fibrous capsule tissue. The smooth device appears to be free of the surrounding tissue with a folded configuration (Figure 5). These findings were further confirmed by SEM imaging. SEM allowed for a closer visualisation at the fibre organisation in the capsules. Fibres surrounding the multiscale porosity device appeared to have disorganised collagenous configuration as they envelope around the rope-coil structures at the device surface. In contrast, the fibrous capsule surrounding the smooth device appeared highly aligned and distinctively layered. The absence of any distinct surface features on the smooth device facilitated the formation of a fibrous capsule, which was highly aligned longitudinally down the length of the device. 

### 3.5. Histological Analysis

**Figure 6:**
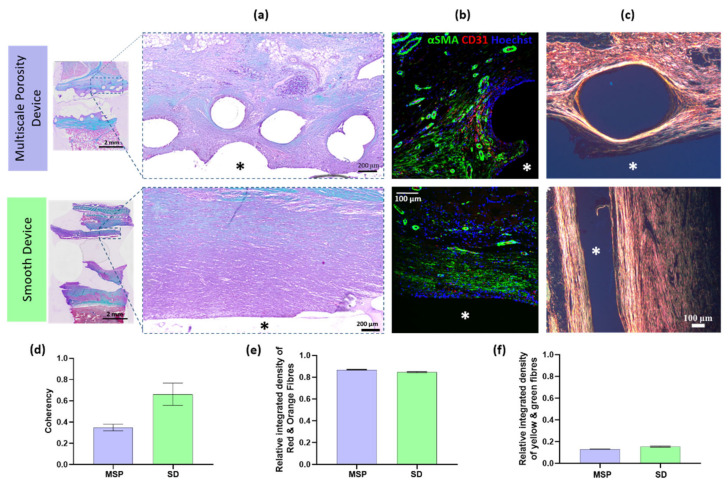
Histological analysis of fibrous capsule formation around multiscale porosity and smooth surfaced devices. (**a**) Masson’s trichrome images of core biopsy taken through device and adjacent surrounding tissue (Scale bar = 2 mm) with magnified representative image of the device space (*) and the fibrous capsule (Scale bar = 200 µm). (**b**) Immunofluorescent images for analysis of myofibroblast density within the fibrous capsule (Hoechst, blue; αSMA, green; CD31, red). Scale bar = 100 µm. (**c**) Polarized Light Microscopy (PLM) images of the fibrous capsules. Scale bar = 100 µm. (**d**) Fibrous capsule coherency measurement using PLM. (**e**) Relative integrated density of red and orange collagen fibres (indicative of mature/permanent collagen deposition). (**f**) Relative integrated density of yellow and green fibres (indicative of immature/active collagen deposition). *n* = 2 per group; data are represented as means ± standard deviation. No significant difference was determined using Kruskal–Wallis tests.

Standard histological analysis was performed on tissue sections cut from the sampling cores. The sections were stained with Masson’s Trichrome, picrosirius red, αSMA, CD31 and Hoechst and then imaged for fibrous capsule analysis. Masson’s trichrome stain provided an overview of the capsule, its size, and its interaction with the implanted device. Specifically, this Masson’s Trichome demonstrated the integration of the surrounding tissue with the multiscale porosity surface features, as imprints are seen within the tissue. Masson’s Trichome also provided qualitative information on collagen structure and orientation. The collagenous capsule appears disorganised with no clear dominant direction of alignment around the multiscale porosity devices while the capsular fibres surrounding the smooth devices demonstrated a largely uniform alignment (Figure 6a). Staining for αSMA allowed for insight into the quantity of myofibroblasts at the site of implantation as they are associated with the over-production of collagen in pro-inflammatory reactions, ultimately regulating tissue contraction and fibrosis [23] and can be correlated to the capsule size [20]. Staining with CD31, an endothelial cell marker, enabled us to rule out αSMA cells associated with blood vessels. Qualitatively this staining gives a nod towards collagen orientation and amounts of active collagen deposition (Figure 6b). Lastly, polarised light microscopy (PLM) of picrosirius red stained tissue is one of the most useful histological methods for analysing collagen organisation and maturity (Figure 6c). This approach has been used for decades in medical research [24]. The picrosirius red stain works by enhancing the birefringent properties of the collagen fibres enabling them to exhibit a spectrum of colours when viewed under polarized light depending on the fibre size and packing density, thus demonstrating a clear orientation of collagen fibres [24,25]. Quantification of the directional uniformity of the collagen fibres demonstrated a higher coherency of the fibres in the capsule around the smooth device than the multiscale porosity device (Figure 6d). Colour threshold segmentation was used to quantify both mature collagen type I fibres (red/orange) and thin, collagen type III-like fibres (green) present during the early phases of remodelling. No obvious differences between device groups were found (Figure 6e,f), with both devices displaying more than 80% mature collagen fibres. 

## 4. Discussion

The present study illustrated, for the first time, the use of DTI for qualitative visualisation and quantitative characterisation of the microstructural organisation of the fibrous capsule formed at the tissue-device boundary with large animal samples. Characterisation of the microstructure and dominant components, such as collagen, within a fibrous capsule has the potential to yield significant insight into tissue integration and the FBR with respect to implanted devices. We used this non-invasive and non-destructive imaging technique to analyse the fibrous capsules surrounding two unique human-sized macroencapsulation devices: the silicone Multiscale Porosity Device and the TPU Smooth Device after implantation in a porcine model for 21 days. Due to the sheer size of these tissue samples, core biopsies were taken to enable traditional histological analysis to be performed. This is a form of stereological analysis that allows accurate representative values to be obtained; however, the downside with this method is the potential to miss unique spatial features within the tissue.

DTI was used to investigate the presence of a fibrous capsule around these two devices with distinct topologies. T1- and T2-weighted images of the two devices yielded no discernible contrast between the presence of fibrous capsule and surrounding tissue. T1- and T2-weighted images have been used for identifying fibrous capsules and granulomas around breast implants [26]; however, the envelope and capsule around the implants typically show low signal intensity [27]. On qualitative inspection of the mean diffusion-weighted images (b = 800 s/mm^2^, 32 directions), a large hyperintense region was apparent around the smooth device that is indicative of restricted diffusion. This contrast was absent surrounding the multiscale porosity device. This qualitative observation was quantified by regionally investigating the MD and FA—specifically in the capsules directly surrounding each device and in the more distally located tissue. 

In the DTI-derived parametric maps, specifically the FA and MD, the regional differences within the tissues became even more apparent. A highly anisotropic capsule not only formed around the smooth device but showed no integration at the tissue-device boundary. In this highly aligned capsule, the diffusivity was decreased compared to the surrounding tissue. Tractography offered a qualitative look at the specific alignment of the microstructural organisation in the fibrous capsule. The fibres aligned longitudinally along the length of the device and showed some circumferential alignment at the lateral edge transition from top to bottom of the device. These non-invasive insights from DTI were further confirmed by traditional histological processing and microCT and SEM imaging. Standard tissue staining using Masson’s Trichrome and myofibroblast stains as well as imaging using SEM and microCT all qualitatively suggested that there appeared to be a difference in collagen organisation. MicroCT highlighted the same separation between device and fibrous capsule seen in DTI, while SEM and PLM confirmed the highly aligned fibre organisation within the fibrous capsule. Coherency measurements of the collagen fibres in PLM images demonstrated increased collagen anisotropy around the smooth device. These measurements agree strongly with the FA results whereby the fibrous capsule around the smooth devices showed a 2-fold increase in anisotropy when compared to more distal tissue.

Conversely, the multiscale porosity device showed no clear delineation of a fibrous capsule, but instead a highly integrated tissue at the tissue-device boundary. While the FA maps of the multiscale porosity device show some areas with increased anisotropy, they are sporadically on one face of the device and very localised. The normalised FA and MD values for the multiscale porosity device were around 1, indicating little difference between the tissue around the device and more distally located tissue. With the same tracking parameters used for the smooth device, tractography showed no coherent organisation around the multiscale porosity device, and this was further confirmed with low coherency measurements from PLM. MicroCT and SEM images of the multiscale porosity device also confirm this highly integrated fibrous capsule around the device. 

Although microCT is a very useful large sample imaging modality for providing context into device tissue interactions, it lacks the quantitative microstructural insight gained by DTI allowing for in-depth tissue analysis in this circumstance. Additionally, while traditional histological processing is the gold standard in identifying the underlying microstructure in tissue, its destructive nature and limited size capabilities make it a less than ideal methodology for fully characterising large scale animal tissue samples. PLM using picrosirius red stained sections enhances the birefringent properties of the collagen fibres enabling quantification of the directional uniformity (coherency) and colour segmentation of fibres [24,25]; however, the FA and tractography together yielded the same insight into the anisotropy of the tissues. Through the work presented in this study, DTI has non-invasively and non-destructively yielded volumetric information on the presence and microstructural organisation of fibrous capsules at the tissue-device boundary. 

This study proved that DTI can not only detect the presence of the fibrous capsule surrounding two different devices but has the capabilities to accurately quantify microstructural differences in the capsules. Although islet macroencapsulation devices were used in this study, these simply act as surrogates for any type of biomaterial or medical device, used for any purpose. The fact that the two devices were composed of different materials merely highlights that this method of imaging is versatile and has a broad range of applications. While both devices in this study were polymer based, devices can also include nonferrous, MRI-safe metals such as titanium. Distortions and signal loss around metallic implants is a well-known problem and methods to mitigate these artifacts, such as favouring spin-echo sequences and increasing readout bandwidth, have been explored. DTI is a non-destructive method for high-resolution imaging without the need for staining or special treatment of the tissue which could be used instead of, or in combination with, extensive histological staining, imaging, and analysis. The potential to non-invasively assess device integration during animal studies could lessen the need for increased numbers of animals as terminal time points could be decreased. Lunney et al. recently highlighted the importance of porcine models as biomedical models due to their similarities in anatomy, physiology, immunology, and genome which proving valuable as models for humans [21]. DTI is particularly useful when analysing these large tissue samples ex vivo, providing the viewer with faster insights into device integration, capsule organisation and unique spatial features that may otherwise be missed when using stereological sampling alone. While fixed samples were investigated in this study, they underwent the same fixation time and storage and future studies would aim to investigate the tissue-device boundary and FBR in vivo. Standard spin-echo DTI faces challenges in vivo due to lengthy scan times and sensitivity to motion artifacts, while faster EPI-based acquisitions could be more sensitive to susceptibility differences between the tissue and device. While the results from this work motivate the use of DTI to monitor the FBR at the tissue-device interface, in vivo clinical translation still requires attention. One recent study performed in vivo DTI at the carotid arteries used read-out segmented EPI combined with pulse and cardiac triggering to acquire images. The biggest limitation to this study was that our sample size was small and prevented us from seeing true significant differences between device types, however we feel the data is still strong enough with an *n* = 2 to demonstrate the potential of this system. We plan to implement this form of capsule interrogation in our future large animal preclinical studies in combination with histological analysis.

While this study focused on the presence and microstructural integration of fibrous capsules from two specific device topographies, the feasibility of using DTI for more general encapsulation of medical devices has been established. The potential to non-invasively ascertain fibre orientation within fibrous capsules has strong implications for understanding and predicting the efficacy of implanted devices. Understanding water diffusion around the device also has the potential to yield valuable insight for devices with drug delivery purposes.

## 5. Conclusions

In summary, we can conclude that DTI is a highly informative technique for analysing fibrous capsule composition surrounding medical devices. It is particularly powerful for analysing the entire specimen whereas standard histology requires sampling of the specimen meaning unique features may be missed contributing to sampling error. It is a non-destructive high quality imaging modality that does not require specialist staining or tissue manipulations and can be used instead of, or in combination with, histological analysis. It accurately measures directional uniformity of collagenous fibrous capsules surrounding human-sized devices and could potentially remove the need for extensive sampling of the tissue thus saving time and resources. Insight gained from DTI derived metrics has the potential to inform decisions on devices transitioning from large scale pre-clinical work to early-stage clinical trials.

## Data Availability

The data presented in this study are available on request from the corresponding author.

## Abbreviations

DTI	Diffusion tensor imaging
ERC	European research council
FA	Fractional anisotropy
FBR	Foreign body response
FEFA	First eigenvector-fractional anisotropy
MD	Mean diffusivity
MicroCT	Micro computed tomography
MRI	Magnetic resonance imaging
PLM	Polarized light Microscopy
SEM	Scanning electron microscopy
TPU	Thermoplastic polyurethane
